# The economic consequences of noncompliance in cardiovascular disease and related conditions: a literature review

**DOI:** 10.1111/j.1742-1241.2007.01683.x

**Published:** 2008-02-01

**Authors:** N Muszbek, D Brixner, A Benedict, A Keskinaslan, Z M Khan

**Affiliations:** 1 United BioSource Corporation London, UK and Budapest, Hungary; 2 University of Utah Salt Lake City, UT, USA; 3 Novartis Pharma AG Basel, Switzerland; 4 Novarits Pharmaceuticals Corporation East Hanover, NJ, USA

## Abstract

**Objectives:**

To review studies on the cost consequences of compliance and/or persistence in cardiovascular disease (CVD) and related conditions (hypertension, dyslipidaemia, diabetes and heart failure) published since 1995, and to evaluate the effects of noncompliance on healthcare expenditure and the cost-effectiveness of pharmaceutical interventions.

**Methods:**

English language papers published between January 1995 and February 2007 that examined compliance/persistence with medication for CVD or related conditions, provided an economic evaluation of pharmacological interventions or cost analysis, and quantified the cost consequences of noncompliance, were identified through database searches. The cost consequences of noncompliance were compared across studies descriptively.

**Results:**

Of the 23 studies identified, 10 focused on hypertension, seven on diabetes, one on dyslipidaemia, one on coronary heart disease, one on heart failure and three covered multiple diseases. In studies assessing drug costs only, increased compliance/persistence led to increased drug costs. However, increased compliance/persistence increased the effectiveness of treatment, leading to a decrease in medical events and non-drug costs. This offset the higher drug costs, leading to savings in overall treatment costs. In studies evaluating the effect of compliance/persistence on the cost-effectiveness of pharmacological interventions, increased compliance/persistence appeared to reduce cost-effectiveness ratios, but the extent of this effect was not quantified.

**Conclusions:**

Noncompliance with cardiovascular and antidiabetic medication is a significant problem. Increased compliance/persistence leads to increased drug costs, but these are offset by reduced non-drug costs, leading to overall cost savings. The effect of noncompliance on the cost-effectiveness of pharmacological interventions is inconclusive and further research is needed to resolve the issue.

**Review Criteria**

Studies quantifying the cost consequences of noncompliance with medication for CVD and related conditions were identified through searches of the MEDLINE, EMBASE and NHS Economic Evaluation databases. A manual search of reference lists from retrieved papers was also performed. Qualitative (e.g. type of evaluation, method of quantifying compliance, source of compliance data) and quantitative (medication possession ratio) data were extracted from the study reports.

**Message for the Clinic**

A review of 23 studies quantifying the cost consequences of noncompliance with medication for CVD and related conditions showed that increased compliance/persistence leads to an increase in the effectiveness of treatment and a decrease in medical events. This results in savings in the overall costs of treating CVD and related conditions. Increased compliance/persistence also appears to reduce cost-effectiveness ratios, but this effect requires further investigation.

## Introduction

Cardiovascular disease (CVD) is responsible for more deaths worldwide than any other condition, and a large proportion of healthcare budgets are spent on its treatment and prevention (1). In the USA, for example, 37% of deaths are caused by CVD, and costs related to the disease are estimated to be $401.3 billion for 2006 (2). Deaths caused by CVD account for 34% of all deaths in Germany, 33% of deaths in England and Wales, 25% of deaths in Spain and 21% of deaths in France (2).

The preventative treatment of CVD aims to control related conditions, such as hypertension, hypercholesterolaemia and diabetes. The worldwide prevalence of hypertension was estimated to be 26% in 2000, and this is predicted to rise to 29% by 2025 (3). The figures are even higher in economically developed countries (e.g. Australia, Canada, Germany, Italy, Japan, Spain, Sweden, the UK and the USA), with an estimated prevalence of 37% and 42% in 2000 and 2025 respectively. Diabetes affects almost 6% of the world's population, and the prevalence of type 2 diabetes is estimated to be 1–12% in Europe and 7–28% in North America (4). According to World Health Organisation (WHO) estimates, hypercholesterolaemia is responsible for 18% of global CVD and 56% of global ischaemic heart disease (5).

Yet, for hypercholesterolaemia, for example, < 50% of those qualifying for lipid-modifying treatment actually receive it (6). Of those who do receive treatment, only about one-third achieve their blood high-density lipoprotein (HDL) goal and < 20% achieve their low-density lipoprotein (LDL) goal (6). A similar pattern of under-treatment is seen in hypertension and diabetes. For example, a recent review of national surveys in hypertension among those aged 35–64 years showed a treatment level ranging from 25% (England) to 32% (Italy). Even among patients receiving treatment, the rate of successful hypertension control ranged from only 18.7% in Spain to 40% in England (7). A retrospective, observational study using data from a General Practitioner prescription database in the UK found even poorer control of blood pressure, with only 14.2% of treated patients achieving guideline-determined blood pressure targets at 1 year (8). Similarly, only approximately 40% of adults with type 2 diabetes achieve the goal recommended by the American Diabetes Association of glycosylated haemoglobin levels lower than 7% (9).

The pharmacological treatment of hypertension, hypercholesterolaemia and diabetes reduces the morbidity and mortality of associated CVD (5,10,11). To be effective, however, treatment must continue, sometimes for life, despite an absence of any obvious symptoms or benefit to the patient. Unfortunately, lack of symptoms in CVD and related conditions is one of the most common reasons for patients discontinuing treatment or not taking the prescribed dose at the required intervals. Studies have shown that poor compliance/persistence with medication is encouraged by the chronic and often asymptomatic nature of hypertension and hypercholesterolaemia (12,13). Poor compliance/persistence can decrease the effectiveness of treatment, leading to treatment failure (11,14,15). This, in turn, leads to an increase in the use of healthcare resources and an increase in overall expenditure (16). Similarly, in diabetic patients, poor compliance/persistence is associated with greater comorbidity, higher hospitalisation rates and higher mortality rates than those in patients who are compliant/persistent with their antidiabetic medication (17). Furthermore, the World Health Organisation has suggested that noncompliance with medication is a common problem that leads to compromised health benefits and serious economic consequences in terms of wasted time, money and uncured disease (18). Thus, noncompliance has been recognised as a serious problem with significant economic consequences. Although studies have investigated the extent of the economic effect of noncompliance, such studies have evaluated different aspects of this effect and have not been designed to present a complete picture.

This review explores the cost consequences of noncompliance with pharmaceutical interventions in hypertension, diabetes, dyslipidaemia and heart failure. The aim is to evaluate the effects of noncompliance on the different types of expenditure, such as drug costs, overall healthcare expenditure and productivity costs, and to investigate the effect it has on the cost-effectiveness of pharmaceutical interventions for CVD and related conditions.

## Methods

### Definitions

Two common measures of compliance are adherence (sometimes used as a synonym for compliance) and persistence. Numerous definitions have been used to describe and measure these parameters. In this review, the definitions of the International Society for Pharmacoeconomics and Outcomes Research (ISPOR) were used, whereby compliance is defined as taking medication as prescribed, on time and at the correct dose, and persistence is defined as the continuing use (in time) of the prescribed therapy (19).

### Searches

Searches for relevant studies were conducted using the MEDLINE and EMBASE databases, and the NHS Economic Evaluation Database (NHSEED). The search terms used were: cardiovascular, hypertens*, hyperlipid*, dyslipid*, blood pressure, diabet*, cost*, economic*, compliance (adherence) and persistence. A manual search of the reference lists from retrieved papers was also performed to identify further relevant studies.

### Selection criteria

Studies were deemed relevant if they were English language, human studies published before February 2007; if they involved patients with CVD or related conditions [hypertension, dyslipidaemia, coronary heart disease (CHD), heart failure or diabetes]; if they examined compliance (adherence) and/or persistence to pharmaceutical interventions (even if the primary objective was not to measure compliance/persistence); and if they provided an economic evaluation or cost analysis and quantified the cost consequences of compliance/persistence. Studies published before 1995 were excluded as results from these earlier studies could not be compared with those from more recent studies because of changes in treatment patterns (specifically the emergence of new treatment options), study methodology and the price of healthcare resources, including drug prices.

Studies were also excluded from analysis if the economic consequence of compliance/persistence was not quantified; if they examined noncompliance with antiplatelets, aspirin, digoxin, insulin, non-pharmaceutical therapies or treatment guidelines; or if they were reviews of earlier (pre 1995) research papers, letters to the editor, commentaries or conference abstracts.

Studies were divided into cost studies and economic evaluations. Cost studies examined the effect of compliance/persistence or compliance-enhancing interventions on the cost of treatment or on productivity costs, while economic evaluations examined changes in the cost-effectiveness of an intervention with different compliance/persistence rates using sensitivity analyses. For the latter, only studies that used a univariate sensitivity analysis of compliance/persistence were selected. When both compliance/persistence rates and costs were examined, but the cost consequences of noncompliance were not studied, the study was excluded.

### Data extraction

Qualitative data extracted from the studies included the country where the study was performed, the type of study (retrospective, prospective, model or based on assumptions), the type of evaluation (cost calculation, cost study, cost-effectiveness, cost-benefit or cost-utility analysis), the disease area, the type of study population, the study setting, the study length, the definitions and methods of quantifying compliance and/or persistence, and the source of compliance/persistence data.

Quantitative data extracted from the studies included the medication possession ratio (MPR), which is the most commonly used measure of compliance, and the rate of persistence. The MPR is defined as the days’ supply of a dispensed prescription divided by the number of days between prescription refills (20). For example, if a patient receives 2 months (60 days) supply of medication and obtains a prescription refill 80 days later, the MPR would be 60 days of supply divided by 80 days until the next refill, which is 0.75 or 75%. Persistence is defined by ISPOR as the accumulation of time from initiation to discontinuation of therapy (measured by time metric), and is usually identified by use of a cut-off point.

### Analysis

Quantitative analysis was not possible because of the different methodology used in the different studies, and difficulties in collating cost data from different countries, where treatment patterns and unit costs vary. Results from the different studies were, therefore, compared descriptively.

## Results

### Study characteristics

Twenty-three studies that analysed the effect of compliance and/or persistence on the cost or cost-effectiveness of treatment were identified. Of these, 10 focused on hypertension, seven on diabetes, one on dyslipidaemia, one on CHD, one on heart failure and three on multiple diseases, including diabetes, hypertension, hypercholesterolaemia, general heart disease and heart failure (Table 1). Seventeen of the studies were cost studies and six were economic evaluations. Most were retrospective in design and were conducted in the USA using administrative claims databases in managed care organisations (Medicare and Medicaid; Table 2). Three Italian retrospective studies used the archives of the Local Health Unit of Ravenna, a decentralised body of the Italian National Health Service, which is responsible for providing health care in the province of Ravenna. Two of these three studies analysed the same data but in a different manner (33,34).

**Table 1:** Studies included in the review

References	Country	Disease	Intervention	Hypothesis/study question concerning compliance
Clark et al. (21)	Canada	Diabetes	ACE inhibitors	Should ACE inhibitors be financed in type Idiabetic necropathy, assuming that cost is amajor barrier to compliance?
Balkrishnan et al. (22)	USA	Diabetes	Antidiabetics	To examine the relationship between healthstatus, adherence, and healthcare costs
Balkrishnan et al. (23)	USA	Diabetes	Oral antidiabetics vs. thiazolidinediones(TZD: pioglitazone & rosiglitazone)	To measure the effect of TZD on healthcarecosts and compliance
Hepke et al. (24)	USA	Diabetes	Insulin or oral hypoglycaemic	To determine whether compliance affectswell-being and the total costs of diabetestreatment
Herman et al. (25)	USA	Diabetes	Prevention of type 2 diabetes with the Diabetes Prevention Program, i.e. lifestyle modification(diet, physical activity) or metformin, 850 mgo.d.	To estimate the cost-utility of the DiabetesPrevention Program
Mahoney (26)	USA	Diabetes	Insulin products and oral antidiabetics	To evaluate the effects of changing theformulary status of diabetes drugs and deviceson compliance and healthcare costs
Shenolikar et al. (27)	USA	Diabetes	Pioglitazone	To compare treatment compliance andhealthcare costs in African Americans and allother races
Urquhart (28)	USA	Hypercholesterolaemia	Cholestyramine (six packets per day) vs.placebo; gemfibrozil vs. placebo	To estimate the economic consequences ofcompliance
Tsuyuki et al. (29)	Canada	Heart failure	Patient support programme (salt and fluidrestriction, weighing, exercise, medication use,knowing when to call physician)	To evaluate the effect of adisease-management programme in heartfailure
Cheng et al. (30)	China	Coronary heartdisease	Statin (atorvastatin or simvastatin) monotherapy	To examine the effects of compliance to statintherapy on direct medical costs for coronaryheart disease
Rizzo & Simons (31)	USA	Hypertension	Antihypertensives	Does noncompliance increase healthcare costs?
Hughes & McGuire (32)	UK	Hypertension	Antihypertensives (ACE inhibitors, beta-blockers,calcium antagonists, diuretics)	To calculate the costs arising from switchingand discontinuing therapy
Degli Esposti (33)[reanalysed inDegli Esposti (34)]	Italy	Hypertension	Antihypertensives	To identify clinical and economic indicators ofpharmacoutilisation of antihypertensives
Mar &Rodriguez-Artalejo (35)	Spain	Hypertension	Antihypertensives	Cost-effectiveness of treatment for arterialhypertension, by age, sex, type of drug andcompliance
Urquhart (36)	USA	Hypertension	Electronic monitoring of compliance	Basic calculation of monitoring for compliance
Degli Esposti (34)[reanalysis ofDegli Esposti (33)]	Italy	Hypertension	Antihypertensives	To identify clinical and economic indicators ofpharmacoutilisation of antihypertensives
Côte et al. (37)	Canada	Hypertension	Pharmacy-based health promotion programmeto improve blood pressure control byimproving the quality of prescribing andadherence to treatment. Pharmacists warned ifpatients non-adherent.	To describe the impact of the programme oncosts and benefits
Taylor & Shoheiber (38)	USA	Hypertension	Amlopidine besylate/benazepril HCl, singlecapsule, fixed dose vs. ACE inhibitor + dihydropyridine calcium-channel blockerseparately	To evaluate the effect of the combinationproduct on compliance and costs
Degli Esposti et al. (39)	Italy	Hypertension	Antihypertensives	To evaluate how long patients remain ondifferent antihypertensives
Rosen et al. (40)	USA	Hypertension	Medicare first-dollar coverage vs. no coverage(current practice) with ACE inhibitor useincreasing from 40% to 60%	To estimate the cost-effectiveness to Medicareof first-dollar (no cost-sharing) coverage ofACE inhibitors (lisinopril) in patients withdiabetes
Rizzo et al. (41)	USA	Multiple – hypertension,heart disease,depression, type 2diabetes	Relevant intervention for the disease inquestion	To evaluate whether drug coverage andcompliance programmes are cost-effectivesaving for employers; how does compliancemodify the cost of treatment?
Plans-Rubió (42)	Spain	Multiple – prevention ofcoronary heart disease(hypertension,hypercholesterolaemia,smoking)	Cholesterol-lowering and antihypertensivedrugs, smoking cessation	How does compliance modifycost-effectiveness?
Sokol et al. (43)	USA	Multiple – diabetes,hypertension,hypercholesterolaemia,congestive heart failure	Cardiovascular and antidiabetic drugs	To evaluate the impact of medical adherence

**Table 2:** Studies according to country, design and type of evaluation

	Based on assumptions	Model		Prospective		Retrospective		
Country	Cost calculation	Cost study	Economic evaluation	Cost study	Economic evaluation	Cost study	Economic evaluation	Total
Canada			1	1			1	3
Italy						3		3
Spain			2					2
UK						1		1
USA	1	1	1	3	1	6		13
China				1				1
Total	1	1	4	5	1	10	1	23

### Compliance and persistence: measurement and results

Three studies in hypertensive patients and one in diabetic patients gave no compliance/persistence rates (26,35–37) and in another two studies, compliance/persistence rates were based on assumptions (21,40). In most studies, persistent patients were considered to be those who continued with the same monotherapy they had been prescribed at the beginning of the study. The three Italian studies (33,34,39) used a cut-off point of 273 days, with patients on treatment for < 273 days being considered non-persistent. Another study (32) used a cut-off point of 26 months. Two other studies defined compliance/persistence as patients continuing on therapy for at least 80% of the prescription period or taking 80% of the prescribed dose (25,31). In some studies, different levels of compliance/persistence were defined according to the percentage of time for which patients took their medication (24,28,31). Only one study considered the timing of doses (30).

The rate of compliance or persistence varied according to the type of study, the patient population, the method of data collection and the technique used to measure compliance/persistence. In studies assessing the cost-consequences of noncompliance, compliance rates were 45–80% in diabetes, 15–35% in hypertension, 51–59% in hypercholesterolaemia and 60–96% in other diseases, such as heart failure and CHD. In CHD, 88% of statins were taken in the prescribed time interval. Persistence rates were measured only in hypertension, and ranged from 63% to 81%.

### Cost studies

Overall healthcare costs may be divided into direct and indirect costs. Direct costs are those relating to goods, services and other resources that are consumed in the provision of an intervention or in dealing with the side effects or other current and future consequences. The resources used can be either medical or non-medical. Thus, direct costs include drug costs, but the major proportion of direct costs is non-drug-related. Indirect costs refer to productivity gains or losses relating to illness or death (44). To calculate the total economic consequences of changes in compliance/persistence, both direct and indirect costs should be considered.

Most studies in this review examined only direct costs and four studies considered only medication costs (28,33,34,39). Indirect costs were considered in one cost study and three economic evaluations (35,37,40,41).

#### Drug costs

The three Italian studies investigated the drug costs associated with different patterns of compliance and persistence with antihypertensives (33,34,39). The average drug costs were highest for patients adding another drug to their therapeutic regimen and lowest for occasional users. The costs were significantly higher for persistent patients who either switched therapies or added another drug to their regimen compared with those who stayed on the same monotherapy throughout the study (Table 3).

**Table 3:** Average drug costs per patient according to the pattern of persistence with antihypertensive medication (34)

Pattern of persistence	Average cost per patient (€)
Same therapy	121.51
Combination	274.69
Switching	182.25
Interruption	65.86
Occasional	32.80

In the later Italian study (39), average drug costs were found to be lower for a combination group consisting of those remaining on the same therapy and those adding another drug to their regimen, than for those who switched drugs. Among the different types of antihypertensives, angiotensin II antagonists were associated with the highest percentage of continuers (41.7%), resulting in high drug costs. Average drug costs were also high for calcium-channel blockers, despite the relatively low continuation rate (26.7%). Conversely, average costs were relatively low for beta-blockers despite the relatively high percentage of continuers (36.9%). Diuretics were associated with the lowest percentage of continuers (25.9%) and the lowest drug costs (Table 4).

**Table 4:** Annual average drug costs per patient for different antihypertensives according to the pattern of persistence (39)

Antihypertensive	Continuers	Switchers	Discontinuers	Whole study cohort
Diuretics	€65.09	€153.10	€8.17	€33.45
Beta blockers	€109.29	€158.73	€22.52	€63.40
Calcium-channel blockers	€234.63	€199.62	€38.24	€104.43
ACE inhibitors	€196.28	€237.53	€34.76	€108.25
Angiotensin II antagonists	€326.16	€268.07	€67.10	€201.53

In another study (28), data from two clinical trials were used to examine compliance and persistence with cholestyramine and gemfibrozil in patients with hypercholesterolaemia. Drug costs for preventing one coronary event and for different levels of compliance and persistence were calculated. The results showed that although drug costs were higher for more compliant/persistent patients, the relative risk of CHD was lower and the drug costs for preventing one coronary event were very similar for the different levels of compliance and persistence.

Estimating drug costs only gives an indication of the effect of noncompliance with the use of different drug types and acquisition prices; it does not provide data on the consequences of noncompliance on other healthcare costs, such as hospitalisation. As a result, even if a given drug or class of drug is associated with a low compliance rate (e.g. diuretics), the increase in drug costs as a result of switching can be offset by a high proportion of generics and thus relatively low drug costs.

#### Direct costs

When other, non-drug costs are taken into account, the results obtained are quite different. The disease-related and all-cause direct healthcare costs in relation to compliance were investigated in a multiple disease study (43). All-cause costs were defined as any healthcare costs over a 1-year period, while disease-related costs were considered to be those associated with the disease only.

**Figure 1:**
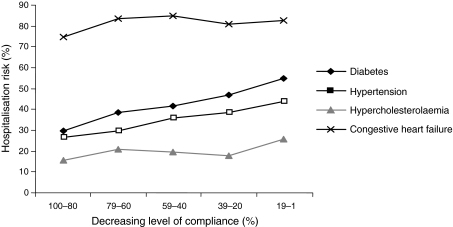
Risk of hospitalisation in relation to the level of compliance for diabetes, hypertension, hypercholesterolaemia and CHF (43)

**Figure 2:**
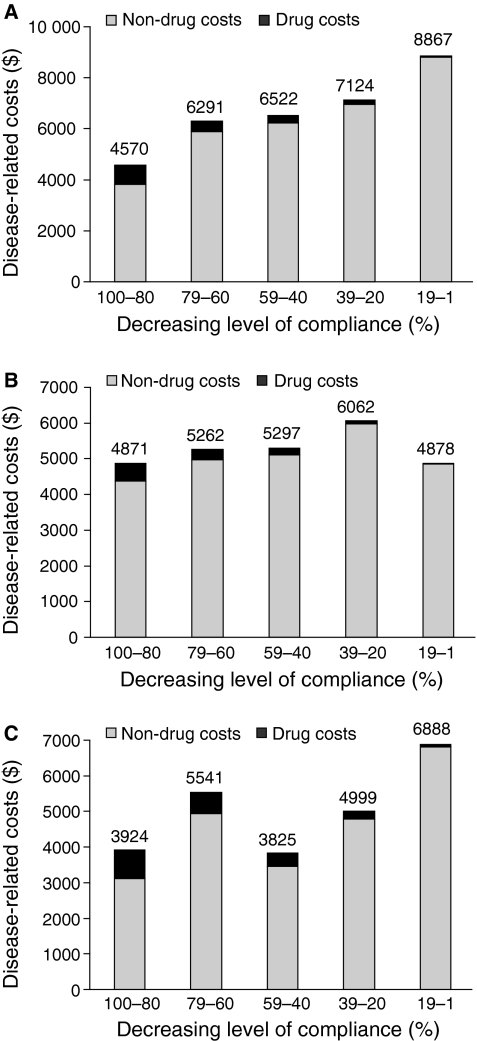
Disease-related healthcare costs in relation to the level of compliance for diabetes (A), hypertension (B) and hypercholesterolaemia (C) (43)

For all-cause costs, a high level (80–100%) of compliance with treatment for diabetes, hypertension and hypercholesterolaemia was associated with significantly lower non-drug medical costs than for lower levels (1–79%) of compliance ($6377 vs. $9363–15,186 for diabetes; $6570 vs. $7658–10,286 for hypertension and $4780 vs. $5509–9849 for hypercholesterolaemia; p < 0.05 for high level of compliance vs. lower levels). As these represent the major proportion of costs, higher levels of compliance with treatment were associated with lower overall healthcare costs, despite high drug costs. In diabetes, overall healthcare costs decreased with increasing compliance, and similar, although nonmonotonic, decreasing trends were seen in hypertension and hypercholesterolaemia. The decrease in healthcare costs with increasing compliance was attributed mainly to a decrease in the risk of hospitalisation, which led to a decrease in non-drug medical costs (Figure 1). Similar associations were seen for disease-related costs (Figure 2). In diabetes and hypercholesterolaemia, higher levels of compliance were associated with lower disease-related costs, despite the high drug costs (p < 0.05 for 80–100% compliance vs. lower levels of compliance), again because of a lower risk of hospitalisation (43). The results for hypertension followed the same pattern but were not statistically significant.

In the above study, no noticeable trends in overall costs were observed for congestive heart failure (CHF) (43). A number of reasons could explain this finding, including a significantly smaller number of patients compared with the other disease areas; high variability in costs; a higher mean chronic disease index in these patients compared with other disease areas; higher hospitalisation costs for CHF patients; higher compliance rates among CHF patients as a result of the greater severity of their disease and little change in the risk of hospitalisation with increasing compliance (Figure 1).

Similarly, a study of compliance with statin monotherapy in patients at high risk of CHD showed that the level of compliance over a 6-month period had no significant effect on direct healthcare costs (30). In this study, however, compliance data were heavily skewed and clustered around 100%, possibly due to the fact that patients who agreed to participate in the study were more motivated to comply with treatment than patients who did not give consent. Furthermore, the study was too short (6 months) to enable the effects of statin therapy on disease control to be properly observed, as the results of a long-term retrospective study in hypertension have shown that persistence decreases significantly between the first and the second year after the start of pharmacological treatment (45), suggesting that a follow-up period of at least 1 or 2 years is required to properly assess the effects of noncompliance. In prospective clinical studies, the drop in persistence may occur even later because of the strict monitoring of patients and their higher motivation to comply with treatment.

The findings in diabetes are supported by the results of other studies in diabetic patients. In one study (22), MPR was found to be the strongest predictor of decreased total annual healthcare costs after controlling for the type of medication and other variables, a 10% increase in MPR being associated with an 8.6% decrease in total annual healthcare costs (p < 0.001). In another study (27), a 10% increase in MPR was associated with a 2% decrease in total annual healthcare costs and a 4% decrease in diabetes-related annual healthcare costs (p < 0.001 and p < 0.01 for total and diabetes-related costs respectively). Another study (24) found a threshold effect, whereby non-drug medical costs increased until the level of compliance reached 20–39% or 40–59%, and then decreased. The decrease was caused by fewer emergency room visits and hospitalisations after a certain threshold of compliance. This threshold effect could have been influenced by the fact that patients with zero per cent compliance included not only non-compliers not filling their prescription as recommended, but also patients whose diabetes was controlled through exercise and diet only, and patients who filled prescriptions under another health plan.

Concern over the rising prevalence and costs of diabetes to a large (23,000 employees) company in the USA led the company to redesign its drug benefit scheme, such that diabetic employees (and their dependants) were required to pay only 10% of the cost for both brand-name and generic antidiabetic medications, rather than 30–50% as before (26). The rationale behind this was that a predictive model had shown that poor compliance was linked to increased healthcare costs, and reducing the cost of treatment to patients would increase compliance, thereby reducing complications and healthcare costs. After 2–3 years, compliance rates had increased, although these were not presented (26). Average total pharmacy costs decreased by 7% and emergency department visits decreased by 26%. Overall, direct healthcare costs per patient decreased by 6% from 2001 to 2003.

In a retrospective study of hypertensive Medicaid patients (31), the highest direct costs were incurred by patients who changed medication by switching or adding another antihypertensive (US$2142), followed by non-persistent patients ($735, p = 0.05 vs. first group) and noncompliant patients ($694, p ≤ 0.001 vs. first group). The lowest costs were seen with persistent patients ($341, p ≤ 0.001 vs. first group). However, the compliance/persistence data in this study were subject to recall bias as they were based on self-reported compliance/persistence. In a UK study, patients switching medication were again found to produce the highest drug costs (£218 vs. £192 for continuers), while continuers produced the lowest hospital costs (£46 vs. £70 for those switching medication) (32).

**Figure 3:**
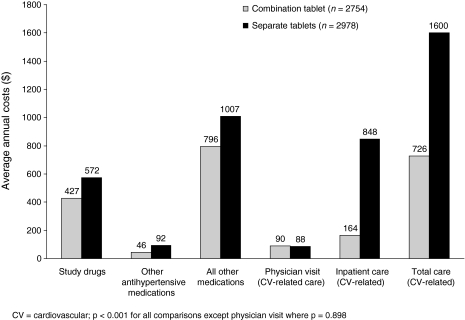
Average annual costs per patient for hypertensive patients taking a combination tablet of an ACE inhibitor and calcium-channel blocker or separate tablets (38). p < 0.001 for all comparisons, apart from physician visit, where p = 0.898

Another study in hypertensive patients compared a fixed-dose tablet, consisting of a combination of an angiotensin converting enzyme (ACE) inhibitor and calcium-channel blocker, with an ACE inhibitor and a calcium-channel blocker taken separately (38). The MPR for the fixed-dose tablet was significantly higher than that for the ACE inhibitor and calcium-channel blocker taken separately (80.8% vs. 73.8%, p < 0.001). Costs (study drugs, other antihypertensives, other drugs, cardiovascular-related inpatient costs and total care costs) were significantly lower in the group receiving the combination tablet than in the group receiving the two drugs separately (Figure 3).

A study of healthcare costs in heart failure (29) found that costs were lower for patients who participated in a support programme than for those who did not participate in the programme, which included salt and fluid restriction, regular weighing and exercise, proper medication use and knowing when to call the physician. After 6 months of follow-up, the average MPR for ACE inhibitors was 86.2% for those participating in the programme and 83.5% for the control group. The difference between the compliance rates was not significant, but this may have been due to the short follow-up (only two refill cycles). Furthermore, frequent contact with the study coordinator in both groups could have influenced the compliance rates. Cardiovascular-related costs with and without the programme were CAN$2017 and CAN$4548, respectively, while overall costs were CAN$3691 and CAN$6154 respectively. The reduction in costs with the patient support programme was attributed to a significant reduction in the total and average length of cardiovascular-related hospital stays (341 days vs. 812 days and 6.4 days vs. 11.6 days, respectively; p = 0.003 for both comparisons) and a significant reduction in cardiovascular-related emergency room visits (20 vs. 49, p = 0.03).

#### Indirect costs

Only one of the cost studies investigated both direct and indirect costs (41). Besides drug costs, this study examined indirect costs in terms of days missed from work. The aim was to calculate the overall cost effects of employer-provided drug coverage and of increasing compliance to 100%. Over a 1-year period with average co-payments of 63% in hypertension, 56% in heart disease and 67% in diabetes, employers acquired $30–46 extra drug costs per employee. Increased compliance resulted in 3.5–16.1 saved work days per employee. Assuming an average wage of $9.32 per hour and fringe benefits of 25%, the benefit from avoiding missed work days was greater than the extra drug costs paid by the employers, resulting in a significant yearly net benefit to the employers ($286–$1475 per employee; Table 5). Assuming that compliance can be increased to 100%, additional drug costs would amount to $16–$27 per employee, while the yearly saving in indirect costs would amount to $191–$962 per employee. However, as this assumption is not realistic, these savings can only be interpreted as upper limits of the potential savings.

**Table 5:** Benefits to the employer of employer-provided drug coverage and increasing compliance to 100% (41)

Disease area and compliance level	Treatment effect (days saved)	Employer costs	Employer savings	Net benefit
**Hypertension**				
Average compliance (37% drug coverage)	3.48	$39	$325	$286
Additional benefit if compliance increased to 100%	2.05	$22	$191	$169
**Heart disease**				
Average compliance (44% drug coverage)	7.28	$46	$679	$633
Additional benefit if compliance increased to 100%	4.46	$27	$416	$389*
**Diabetes**				
Average compliance (33% drug coverage)	16.15	$30	$1505	$1475
Additional benefit if compliance increased to 100%	10.32	$16	$962	$946*

* Calculated from the data in the study and not equivalent to the one given in the published report ($370 and $932).

### Economic evaluations

Of the six economic evaluations, two assessed the cost-effectiveness of drug coverage using the assumption that high drug costs are a major barrier to compliance/persistence and that higher coverage would increase compliance and persistence with therapy; two analysed health promotion programmes and two assessed the economic effects of compliance and persistence in sensitivity analyses. All of the studies apart from two (25,37) were based on decision models.

#### Cost-effectiveness of drug coverage

A Canadian study (21) evaluated the effect of provincial payment for ACE inhibitors in patients with type I diabetes with microalbuminuria. The cost-utility model ran for 21 years and incorporated direct medical costs. The long-term compliance rates were assumed to be 50% without payment and 67% with payment. Based on a previous report (46), creatinine clearance was assumed to decline at a rate of 11% per year in patients who complied with treatment and at a rate of 17% per year in noncompliant patients. Based on these assumptions, a compliance rate of 34% resulted in an increase in quality adjusted life-years (QALYs) of 0.147 and a decrease in annual direct medical costs of CAN$849. Thus, the provincial payment was dominant (i.e. both more effective and cost saving). This result was sensitive to the assumptions for compliance, drug costs and survival times. Provincial payment was the dominant strategy, if compliance increased to at least 66%, and remained under the threshold of CAN$20,000, if compliance increased to at least 63%. If drug costs were reduced by 50%, provincial payment was the dominant strategy with a compliance rate of 58% or more.

The above study had many limitations. Namely, compliance and its effect on efficacy were based on assumptions; survival was calculated for all diabetes patients (type I and II disease); dialysis costs were probably underestimated and the life span of compliant patients was assumed to be similar to that of noncompliant patients. However, similar conclusions were reached in a study of Medicare diabetic patients (40). In this study, the lifetime cost of diabetes in the drug-benefit scheme was US$110,590 and $123,973 for compliant and noncompliant patients, respectively, while the QALYs were 8.82 and 7.67 respectively. In the no-coverage scheme, corresponding costs were $107,914 and $123,973 respectively. Assuming an increase in compliance of 50% (from 40% to 60%) with ACE inhibitors compared with no coverage, first-dollar coverage proved to be the dominant strategy, resulting in an increase in effectiveness and a decrease in costs. Such dominance was observed in 91% of the simulations, while the cost-effectiveness ratio for first-dollar coverage was < $20,000 in 99% of cases. Besides the higher ACE inhibitor costs, future unrelated healthcare costs were also taken into account. These costs were offset by the medical events prevented.

#### Health promotion programmes

The economic effects of compliance were evaluated in sensitivity analyses of data from a clinical trial investigating the prevention of type 2 diabetes in patients with impaired glucose tolerance (25). The Diabetes Prevention Program consisted of lifestyle modification (diet, physical activity), or treatment with metformin, 850 mg once daily, or placebo. The incremental cost-effectiveness ratio (ICER) was $1124/QALY for lifestyle intervention and $31,300/QALY for metformin. At the end of the trial, 72% of metformin and 77% of placebo patients had taken at least 80% of the prescribed dose. Assuming that compliance would decrease after the third year of treatment resulting in a 20–50% reduction in treatment effectiveness, the ICER would increase to $3100–7900/QALY for lifestyle intervention and to $38,000–52,600/QALY for metformin.

A controlled trial evaluated a Canadian pharmacy-based health promotion programme in hypertension (37). The programme aimed to improve blood pressure control by improving the quality of prescribing and compliance with treatment. Although no compliance results were reported, antihypertensive drug refills were higher in the intervention group than in the control group. Assuming that the two groups required the same average number of refills, compliance could be considered to be higher in the intervention group. Programme costs were CAN$30.68 per participant. Compared with the control group, indirect costs for the group participating in the programme were significantly increased (by CAN$40.7 per participant, p < 0.001), while direct costs significantly decreased (by CAN$331.3, p = 0.032), resulting in a decrease in overall costs of CAN$290.6 (p = 0.06). The net benefits of the programme for the 9-month period of the study were CAN$264.78 per participant. The internal validity of this study is questionable, however, because of the significant difference between the two groups with respect to income (more low income subjects in the group participating in the programme) and means of transportation (more walkers in the programme group).

#### Sensitivity analyses

A Spanish study evaluated the different parameters influencing the cost-effectiveness of antihypertensives in patients with stage I and II arterial hypertension (35). Direct non-medical costs and indirect costs were incorporated into some of the scenarios in addition to direct medical costs. The cost of an additional QALY amounted to €3307–34,516. The cost-effectiveness ratio decreased with increasing age and was less in men than in women. The inclusion of travel and productivity costs increased the cost-effectiveness ratio by 30% in women and by 35% in men. Assuming a linear relationship between compliance and efficacy, a decrease in compliance of 50% resulted in an increase in ICER of 30–50%. This increase was greater in older patients and was greater in men than women.

Smaller effects were reported in another Spanish study investigating the primary prevention of CHD (42). In this study, the effect of noncompliance with hydrochlorothiazide and lovastatin was calculated only for patients with mild hypertension (diastolic blood pressure 95–104 mm Hg) and hypercholesterolaemia (cholesterol level > 7.7 mmol/l). The results showed that a 10% decrease in compliance produced only a marginal change in the ICER: for treatment with lovastatin in patients with hypercholesterolaemia, a 10% decrease in compliance increased the ICER from $US34,415 to $34,712 per life-year gained. The corresponding figure for treatment with hydrochlorothiazide in patients with mild hypertension was an increase from $11,906 to $12,025 per life-year gained.

## Discussion

Noncompliance in CVD and related conditions is an important issue because of the chronic and often asymptomatic nature of such disease, resulting in poor disease control and long-term adverse consequences. This review confirms that noncompliance also has a significant effect on costs. However, the effect differs depending on the type and the range of costs taken into account. This effect, in turn, influences the cost-effectiveness of pharmaceutical interventions for CVD and related conditions.

### Effect of noncompliance on cost

Noncompliance appears to have a significant effect on the costs of treatment. High compliance and persistence lead to an increase in drug costs (28,33,34,39), while low compliance/persistence is associated with increased medical events and hence more physician visits and hospital admissions, and longer hospital stays (16,43). The increased use of non-drug resources with lower levels of compliance and persistence results in higher overall costs in diabetes (22,24,26,27,43), hypertension (31,32,43) and hypercholesterolaemia (43). However, the results of studies in CHD and heart failure are inconclusive (29,30,43). Noncompliance can also lead to lost productivity because of a higher number of days missed from work (41).

While the correlation between compliance/persistence and inpatient costs seems to be clear, the relationship between physician visits and compliance/persistence is two-sided. For hypertension, controlled blood pressure reduces the number of physician visits and hence medical costs. However, physician visits can also increase compliance/persistence by encouraging patients to comply with their treatment (39).

### Effect of compliance on cost-effectiveness

Higher compliance/persistence rates appear to lower the cost-effectiveness ratio in diabetes (21,25,40) and hypertension (35,37,42). However, the studies reviewed rarely estimated these effects specifically, and if they assessed the effects of compliance/persistence quantitatively, they made assumptions (e.g. a linear relationship between compliance/persistence and effectiveness) to facilitate calculations (35).

Although some of the economic evaluations incorporated noncompliance into the cost-effectiveness calculations, they did not report the numerical effects of changing the compliance/persistence rate. Rather, they simply reported whether or not it had an important effect. Thus, the results of these studies were inconclusive. For example, in a study to determine the cost-effectiveness of statins in the prevention of CHD, improved compliance did not change the order of treatments in terms of cost-effectiveness, but only changed the overall cost-effectiveness of statin therapy (47). Another study in hypertension found that although the cost of achieving blood pressure control was sensitive to compliance, the overall costs of antihypertensive treatment were not (48). Similarly, in type 2 diabetes, the marginal cost-effectiveness ratio was not sensitive to a decrease in discontinuation rates (49).

### Fixed-dose combinations

Fixed-dose combinations of different drugs may help to increase patient compliance/persistence in cases where more than one type of drug is being taken. In hypertension, such combinations have the potential to improve disease control and avoid adverse medical events, thus increasing effectiveness and lowering non-drug medical costs (38). The improvement in disease control with the use of fixed-dose combinations can reduce the number of hospitalisations and physician visits, leading to a decrease in overall healthcare costs and an improvement in cost-effectiveness (38). Most studies assessing the cost consequences of fixed-dose combinations do not include estimates of compliance/persistence. However, retrospective studies have shown that fixed-dose combinations in hypertension can lead to better compliance and persistence (50–52). This, in turn, leads to better health outcomes and fewer medical events (53). The results of one study in diabetes (26) also suggest that increased use (from 9% to 22%) of fixed-dose combinations contributes to decreased healthcare costs.

### Factors influencing the economic effects of compliance

In most of the studies reviewed, the time interval analysed was 2 years or less. This may have influenced the results, since the time frame influences the effect of noncompliance. In the short term, good compliance/persistence is associated with an increase in the amount of medication taken, thus leading to an increase in drug costs. In the long term, however, medical events are avoided. The point where the savings from the non-drug costs offset the extra drug costs (i.e. where increasing compliance/persistence further would be cost-saving) depends on the drug costs, the avoided events and their costs, and how far into the future these events take place. The latter is determined by the course, nature and severity of the disease.

In addition to the time interval, the type of healthcare system and reimbursement scheme can also influence the results. In healthcare systems involving significant patient co-payment, better compliance/persistence increases out-of-pocket payments. Hence, patients bear the higher financial burden, while savings are realised by the third-party payer (e.g. NHS or insurance company). This could reduce the patient incentive to be compliant, particularly in diseases where the consequences of noncompliance are realised in the very distant future, such as hypertension (1). However, in disease areas where poor compliance/persistence results in immediate or near deterioration of health, the incentive to be compliant is greater. In healthcare systems involving insignificant co-payment, the short-term extra costs and the long-term savings are realised within the same organisation, avoiding the higher financial burden of better compliance/persistence. This could encourage patients to be more compliant, and payers to consider the long-term savings that can be achieved with higher compliance/persistence.

### Considerations for future cost studies and economic evaluations

Retrospective measurement with the aid of claims or pharmacy databases is a comparatively easy, precise and quick way of measuring compliance/persistence. However, possession of medication does not necessarily indicate consumption: hoarding and skipping of medication can occur and the timing of doses cannot be examined. It is also difficult to know which drug is responsible for the observed effects because of the high number of add-on therapies and because of switching between different drugs. In addition, retrospective collection of data does not allow for the selection of patients, so different treatment groups could differ significantly in their characteristics. Prospective collection of data (e.g. alongside clinical trials) allows greater flexibility in the selection of patients, control groups and compliance/persistence measures. However, regular meetings with study investigators, the greater attention devoted to compliance/persistence and the selection of specific patients could bias the results, producing higher compliance/persistence rates than those observed in real life. If feasible, a prospective, real-world, observational study could represent a more realistic picture of what happens in a real-world, usual-care setting.

Most of the studies included in this review used retrospective data. Accordingly, the most common measure of compliance used in the different studies was the MPR. However, compliance rates could not be compared across studies because of the different patient populations. As most of the retrospective studies used the claims databases of managed care organisations, such as Medicare and Medicaid, the applicability of the results may be limited to settings where third party payers are responsible for distributing health care. Further research is needed to confirm that increased compliance and persistence are associated with cost savings. Today, payers may relate increased compliance and persistence to increased short-term costs because of the impact on their budget. However, although drug costs alone may slightly increase, these costs are often off-set by reduced non-drug costs, which lead to overall cost savings.

### Future research

The association between patient compliance/persistence with medication and disease outcomes, such as cardiovascular or all-cause morbidity and mortality, has rarely been evaluated outside of the clinical trial setting. A recent publication from investigators at Colorado's Kaiser Permanente showed higher risks for all-cause hospitalisation and mortality in patients with diabetes who were non-optimally compliant with statins, antihypertensives and oral hypoglycaemic agents (17). Similar analyses are needed to evaluate the relationship between compliance/persistence with antihypertensives and blood pressure outcomes in patients with hypertension but without significant comorbidities, such as diabetes.

Future research should focus on long-term, real-world, longitudinal studies to measure the actual costs and savings associated with increased compliance and persistence, and the impact on positive health outcomes, such as improved blood pressure, lipid levels or glycosylated haemoglobin levels. The most significant limitation of retrospective analyses of administrative claims databases is that the impact of compliance on clinical outcomes across the different drug classes is not obtainable. These limitations can be overcome using a retrospective electronic medical record database. However, as drug use is recorded by prescription order, compliance data is lacking. Another alternative is to conduct such research using databases with access to both prescription refill information and information on outcomes, such as blood pressure. The Veterans Health Administration (VHA) health information system may serve such a purpose. Results from these studies would provide physicians and decision makers with additional insights into the various factors that impact on treatment effectiveness, and may lead to a paradigm shift with increased focus on the benefits of compliance and persistence rather than on drug costs alone.

An ongoing study in the VHA is addressing the question of whether poor compliance with antihypertensive therapies is associated with an increase in hospitalisation and mortality rates or failure to improve clinical disease measures, such as blood pressure outcomes. Data from electronic health records and pharmacy records will be collected from the date of the first prescription for an antihypertensive. The study timeline will include a 1-year compliance assessment period and a 12- to 18-month outcomes assessment period. The primary outcome of the analysis will be a composite end-point of all-cause hospitalisation or death occurring during the outcomes assessment period. Secondary outcomes will include cardiovascular hospitalisation, cardiovascular death, and achievement of blood pressure goals based on recommended levels for patients with and without diabetes, during the outcomes assessment period. It is anticipated that the outcomes of this study will lend support to the outcomes obtained from the current review of retrospective studies that are largely dependent on administrative claims databases and lack clinical outcomes data.

## Conclusions

Noncompliance and non-persistence are significant problems in the management of CVD and related conditions, such as diabetes. The increased drug use associated with higher compliance and persistence is associated with an increase in drug costs. These costs are particularly high for patients switching therapies. However, better compliance/persistence increases the effectiveness of treatment, leading to a decrease in future adverse medical events. Fewer medical events results in lower non-drug costs and, as the majority of healthcare costs are non-drug costs, these offset the higher drug expenditures in the long term, such that a 10% increase in compliance results in a 2–9% decrease in total annual healthcare costs.

Higher compliance/persistence rates also appear to reduce cost-effectiveness ratios, one study showing that a 20% decrease in compliance increased the ICER by $6700/QALY. However, most studies included in this review failed to investigate the extent of the effect, partly because of a lack of understanding about the relationship between compliance/persistence and effectiveness. Thus, the effect of compliance/persistence on cost-effectiveness is currently inconclusive. Further research into the relationship between effectiveness, compliance/persistence and cost-effectiveness is required to resolve the issue.

## References

[1] Peterson AM, McGhan WF (2005). Pharmacoeconomic impact of non-compliance with statins. Pharmacoeconomics.

[2] Thom T, Haase N, Rosamond W (2006). Heart disease and stroke statistics – 2006 update: a report from the American Heart Association Statistics Committee and Stroke Statistics Subcommittee. Circulation.

[3] Kearney PM, Whelton M, Reynolds K (2005). Global burden of hypertension: analysis of worldwide data. Lancet.

[4] Adeghate E, Schattner P, Dunn E (2006). An update on the etiology and epidemiology of diabetes mellitus. Ann N Y Acad Sci.

[5] World Health Organization (WHO) The World Health Report 2002: Reducing Risks, Promoting Healthy Life.

[6] Third Report of the National Cholesterol Education Program (NCEP) (2002). Expert Panel on Detection, Evaluation, and Treatment of High Blood Cholesterol in Adults (Adult Treatment Panel III) final report. Circulation.

[7] Wolf-Maier K, Cooper RS, Kramer H (2004). Hypertension treatment and control in five European countries, Canada, and the United States. Hypertension.

[8] Walley T, Duggan AK, Haycox AR, Niziol CJ (2003). Treatment for newly diagnosed hypertension: patterns of prescribing and antihypertensive effectiveness in the UK. J R Soc Med.

[9] Saaddine JB, Cadwell B, Gregg EW (2006). Improvements in diabetes processes of care and intermediate outcomes: United States, 1988-2002. Ann Intern Med.

[10] Stratton IM, Adler AI, Nell HA (2000). Association of glycaemia with macrovascular and microvascular complications of type 2 diabetes (UKPDS 35): prospective observational study. BMJ.

[11] Halpern MT, Khan ZM, Schmier JK (2006). Recommendations for evaluating compliance and persistence with hypertension therapy using retrospective data. Hypertension.

[12] Ambrosioni E (2001). Pharmacoeconomics of hypertension management: the place of combination therapy. Pharmacoeconomics.

[13] Hughes DA, Bagust A, Haycox A (2001). The impact of non-compliance on the cost-effectiveness of pharmaceuticals: a review of the literature. Health Econ.

[14] Simpson SH, Eurich DT, Majumdar SR (2006). A meta-analysis of the association between adherence to drug therapy and mortality. BMJ.

[15] Bramley TJ, Gerbino PP, Nightengale BS (2006). Relationship of blood pressure control to adherence with antihypertensive monotherapy in 13 managed care organizations. J Manag Care Pharm.

[16] Skaer TL, Sclar DA, Robison LM (1996). Noncompliance with antihypertensive therapy. Economic consequences. Pharmacoeconomics.

[17] Ho PM, Rumsfeld JS, Masoudi FA (2006). Effect of medication nonadherence on hospitalization and mortality among patients with diabetes mellitus. Arch Intern Med.

[18] World Health Organization (WHO) (2003). Adherence to Long Term Therapies: Evidence for Action.

[19] Medication Compliance and Persistence Special Interest Group (MCP) Accomplishments.

[20] Theibaud P, Patel B, Nichol M, Berenbeim D (2005). The effect of switching on compliance and persistence: the case of statin treatment. Am J Manag Care.

[21] Clark WF, Churchill DN, Forwell L (2000). To pay or not to pay? A decision and cost-utility analysis of angiotensin-converting-enzyme inhibitor therapy for diabetic nephropathy. CMAJ.

[22] Balkrishnan R, Rajagopalan R, Camacho FT (2003). Predictors of medication adherence and associated health care costs in an older population with type 2 diabetes mellitus: a longitudinal cohort study. Clin Ther.

[23] Balkrishnan R, Rajagopalan R, Shenolikar RA (2004). Healthcare costs and prescription adherence with introduction of thiazolidinedione therapy in Medicaid type 2 diabetic patients: a retrospective data analysis. Curr Med Res Opin.

[24] Hepke KL, Martus MT, Share DA (2004). Costs and utilization associated with pharmaceutical adherence in a diabetic population. Am J Manag Care.

[25] Herman WH, Hoerger TJ, Brandle M (2005). The cost-effectiveness of lifestyle modification or metformin in preventing type 2 diabetes in adults with impaired glucose tolerance. Ann Intern Med.

[26] Mahoney JJ (2005). Reducing patient drug acquisition costs can lower diabetes health claims. Am J Manag Care.

[27] Shenolikar RA, Balkrishnan R, Camacho FT (2006). Comparison of medication adherence and associated health care costs after introduction of pioglitazone treatment in African Americans versus all other races in patients with type 2 diabetes mellitus: a retrospective data analysis. Clin Ther.

[28] Urquhart J (1999). Pharmacoeconomic consequences of variable patient compliance with prescribed drug regimens. Pharmacoeconomics.

[29] Tsuyuki RT, Fradette M, Johnson JA (2004). A multicenter disease management program for hospitalized patients with heart failure. J Card Fail.

[30] Cheng CWR, Chan JCN, Tomlinson B (2006). Factors associated with healthcare utilization costs for statin therapy – a pilot study in Hong Kong. Int J Clin Pharmacol Ther.

[31] Rizzo JA, Simons WR (1997). Variations in compliance among hypertensive patients by drug class: implications for healthcare costs. Clin Ther.

[32] Hughes D, McGuire A (1998). The direct costs to the NHS of discontinuing and switching prescriptions for hypertension. J Hum Hypertens.

[33] Degli Esposti E, Sturani A, Degli Esposti L (2001). Pharmacoutilization of antihypertensive drugs: a model of analysis. Int J Clin Pharmacol Ther.

[34] Degli Esposti E, Sturani A, Di Martino M (2002). Long-term persistence with antihypertensive drugs in new patients. J Hum Hypertens.

[35] Mar J, Rodriguez-Artalejo F (2001). Which is more important for the efficiency of hypertension treatment: hypertension stage, type of drug or therapeutic compliance?. J Hypertens.

[36] Urquhart J (2001). Some economic consequences of noncompliance. Curr Hypertens Rep.

[37] Côte I, Gregoire JP, Moisan J (2003). A pharmacy-based health promotion program in hypertension: cost-benefit analysis. Pharmacoeconomics.

[38] Taylor AA, Shoheiber O (2003). Adherence to antihypertensive therapy with fixed-dose amlodipine besylate/benazepril HCl versus comparable component-based therapy. Congest Heart Fail.

[39] Esposti LD, Di Martino M, Saragoni S (2004). Pharmacoeconomics of antihypertensive drug treatment: an analysis of how long patients remain on various antihypertensive therapies. J Clin Hypertens (Greenwich).

[40] Rosen AB, Hamel MB, Weinstein MC (2005). Cost-effectiveness of full Medicare coverage of angiotensin-converting enzyme inhibitors for beneficiaries with diabetes. Ann Intern Med.

[41] Rizzo JA, Abbott TA, Pashko S (1996). Labour productivity effects of prescribed medicines for chronically ill workers. Health Econ.

[42] Plans-Rubió P (1998). Cost-effectiveness analysis of treatments to reduce cholesterol levels, blood pressure and smoking for the prevention of coronary heart disease: evaluative study carried out in Spain. Pharmacoeconomics.

[43] Sokol MC, McGuigan KA, Verbrugge RR (2005). Impact of medication adherence on hospitalization risk and healthcare cost. Med Care.

[44] (1996). Cost-effectiveness in Health and Medicine.

[45] Van Wijk BL, Klungel OH, Heerdink ER, de Boer A (2005). Rate and determinants of 10-year persistence with antihypertensive drugs. J Hypertens.

[46] Lewis EJ, Hunsicker LG, Bain RP, Rohde RD, for the Collaborative Study Group (1993). The effect of angiotensin-enzyme inhibition on diabetic nephropathy. N Engl J Med.

[47] Huse DM, Russell MW, Miller JD (1998). Cost-effectiveness of statins. Am J Cardiol.

[48] Ramsey SD, Neil N, Sullivan SD (1999). An economic evaluation of the JNC hypertension guidelines using data from a randomized controlled trial. Joint National Committee. J Am Board Fam Pract.

[49] Golan L, Birkmeyer JD, Welch HG (1999). The cost-effectiveness of treating all patients with type 2 diabetes with angiotensin-converting enzyme inhibitors. Ann Intern Med.

[50] Plauschinat CA, Nguyen AB, Frech FH (2004). Patient compliance and persistence with combination valsartan/hydrochlorothiazide therapy versus hydrochlorothiazide therapy.

[51] Dezii CM (2000). A retrospective study of persistence with single-pill combination therapy vs. concurrent two-pill therapy in patients with hypertension. Manag Care.

[52] Sturkenboom MCJM, Picelli G, Dieleman JP (2005). Patient adherence and persistence with antihypertensive therapy: one-versus two-pill combinations. J Hypertens.

[53] DiMatteo MR, Giordani PJ, Lepper HS (2002). Patient adherence and medical treatment outcomes: a meta-analysis. Med Care.

